# Supplementary material for: Prevalence of Eating Disorders and Disordered Eating Behaviours amongst Adolescents and Young Adults in Saudi Arabia: A Systematic Review
Source: Nutrients. 2023 Nov 1;15(21):4643. doi: 10.3390/nu15214643 (PMC10649920; doi:10.3390/nu15214643)
Supplement: Supplementary file 1 [file nutrients-15-04643-s001.zip › S1.Search Strategies.pdf]

Supplementary Materials. S1 Databases Search Strategy: Ovid MedLine, APA PsycInfo, Scopus, Web of Science, Proquest via ASSIA.

Ovid MEDLINE(R)

#	Query	Results from 6 February 2022
1	(adolescent* or teen* or teenager* or youth*).mp. [mp=title, abstract, original title, name of substance word, subject heading word, floating sub-heading word, keyword heading word, organism supplementary concept word, protocol supplementary concept word, rare disease supplementary concept word, unique identifier, synonyms]	**2,288,433**
2	(eating disorder or disordered eating or feeding disorder or appetite disorder or anorexia nervosa or bulimia nervosa or binge eating or disturbed eating or weight control or purging).mp. [mp=title, abstract, original title, name of substance word, subject heading word, floating sub-heading word, keyword heading word, organism supplementary concept word, protocol supplementary concept word, rare disease supplementary concept word, unique identifier, synonyms]	**43,525**
3	Saudi Arabia.mp. or Saudi Arabia/	**27,874**
4	1 and 2 and 3	**14**

APA PsycInfo

#	Query	Results from 6 February 2022
1	(adolescent* or teen* or teenager* or youth*).mp. [mp=title, abstract, heading word, table of contents, key concepts, original title, tests & measures, mesh word]	**551,981**
2	(eating disorder or disordered eating or feeding disorder or appetite disorder or anorexia nervosa or bulimia nervosa or binge eating or disturbed eating or weight control or purging).mp. [mp=title, abstract, heading word, table of contents, key concepts, original title, tests & measures, mesh word]	**40,856**
3	Saudi Arabia.mp.	**2,652**
4	1 and 2 and 3	1

Scopus

*adolescent** OR *teen** OR *teenager** OR *youth** AND *eating* AND *disorder* OR *disor dered* AND *eating* OR *feeding* AND *disorder* OR *appetite* AND *disor-*

der OR *anorexia* AND *nervosa* OR *bulimia* AND *nervosa* OR *binge* AND *eating* OR *disturbed* AND *eating* OR *weight* AND *control* OR *purging* AND *saudi* AND *arabia*

Web of Science

((ALL= (adolescent* or teen* or teenager* or youth*)) AND ALL=(eating disorder or disordered eating or feeding disorder or appetite disorder or anorexia nervosa or bulimia nervosa or binge eating or disturbed eating or weight control or purg-ing)) AND ALL=(Saudi Arabia)

ProQuest via ASSIA

(adolescent* or teen* or teenager* or youth*) AND (eating disorder or disordered eating or feeding disorder or appetite disorder or anorexia nervosa or bulimia nervosa or binge eating or disturbed eating or weight control or purging) AND (Saudi Arabia)
