# Supplementary material for: Prevalence of Eating Disorders and Disordered Eating Behaviours amongst Adolescents and Young Adults in Saudi Arabia: A Systematic Review
Source: Nutrients. 2023 Nov 1;15(21):4643. doi: 10.3390/nu15214643 (PMC10649920; doi:10.3390/nu15214643)
Supplement: Supplementary file 1 [file nutrients-15-04643-s001.zip › S2. JBI Quality Appraisal Table.pdf]

No.	Authors & Year	JBI quality assessment criteria's								Quality Assessment Score
		Q1	Q2	Q3	Q4	Q5	Q6	Q7	Q8	
#1	AlHazmi & AlJohani (2019)	N	Y	Y	Y	NA	NA	Y	Y	5
#2	Albrahim et al (2019)	Y	Y	Y	Y	NA	NA	Y	Y	6
#3	Alwosaifer et al (2018)	Y	Y	Y	Y	NA	NA	Y	Y	6
#4	Tomar & Antony (2021)	Y	Y	Y	Y	NA	NA	Y	Y	6
#5	Al-Qahtani & Al-Harbi (2020)	Y	Y	Y	Y	NA	NA	Y	Y	6
#6	Al-Subaie (2000)	Y	U	Y	Y	NA	NA	Y	Y	5
#7	Almuhlafi et al (2018)	N	Y	Y	Y	NA	NA	Y	Y	5
#8	Alsubaie et al (2019)	N	Y	Y	Y	NA	NA	Y	Y	5
#9	Fatima & Ahmed (2018)	Y	Y	Y	Y	NA	NA	Y	Y	6
#10	Ghafouri et al (2021)	Y	Y	Y	Y	NA	NA	Y	Y	6
#11	Allihaibi (2015)	N	Y	Y	U	NA	NA	Y	Y	4
#12	Fallatah et al (2015)	Y	Y	Y	Y	NA	NA	Y	Y	6
#13	Loni et al (2022)	Y	Y	Y	Y	NA	NA	Y	Y	6
#14	ElShikieri (2022)	Y	Y	Y	Y	NA	NA	Y	Y	6

Note: Y - Yes, N - No, U – Unclear, NA-Not applicable
Q1= Were the criteria for inclusion in the sample clearly defined?
Q2= Were the study subjects and the setting described in detail?
Q3= Was the exposure measured in a valid and reliable way?
Q4= Were objective, standard criteria used for measurement of the condition?
Q5= Were confounding factors identified?
Q6= Were strategies to deal with confounding factors stated?
Q7= Were the outcomes measured in a valid and reliable way?
Q8= Was appropriate statistical analysis used?

Supplementary Material. S2 JBI Quality Assessment Tool for the Quality Appraisal of the Selected Records
